# Overlapping attempts to falsify and Darwinize the Gaia hypothesis

**DOI:** 10.1098/rstb.2024.0087

**Published:** 2025-08-07

**Authors:** Richard Boyle

**Affiliations:** ^1^ Global Systems Institute, University of Exeter, Exeter, England, UK

**Keywords:** Gaia hypothesis, It's-the-song-not-the-singers theory, persistence selection, habitability, biogeochemistry

## Abstract

Evidence for life’s manifestation at the planetary scale is compelling, but arguably interpretable in terms of interactive life-environment coevolution rather than homeostatic regulation. Here, I argue that a substantive and testable Gaia hypothesis must invoke an entity that is planetary in scale, habitability-promoting, and life-specific. This necessitates a resolution to Gaia’s perennial 'Darwinization problem’: there is no *a priori* reason to expect natural selection within biological populations to favour genotypes conducive to global geochemical/climatic habitability. Three potential routes are outlined for such a resolution, differing in terms of whether the Gaia hypothesis is perceived as: (i) probably incorrect and therefore without need of Darwinization; (ii) potentially correct but associated with some life-induced habitability-promoting influence unconnected to natural selection; and (iii) potentially correct but incomplete without a natural-selection-focused description of habitability. Option (i) downplays the relevance of planetary scale properties to both terrestrial habitability and extra-terrestrial life detection, whereas (ii) sidesteps the Darwinization problem by unacceptably reducing Gaia’s life-specificity, making (iii) preferable because it uniquely preserves Gaia’s core properties. However, this requires that Doolittle’s recent 'It’s-the-song-not-the-singers’ theory be supplemented with a biogeochemical/climatic description of Darwinian fitness that leads to unique predictions. I summarize preliminary attempts to derive such a description.

This article is part of the discussion meeting issue ‘Chance and purpose in the evolution of biospheres’.

## 1. Defining the problem

In his 1981 review [1, p. 59-60] of James Lovelock’s first book-length articulation of the Gaia hypothesis [2], Ford Doolittle wrote:

*It is not novel to suggest that life has profoundly changed the Earth, but it is novel, and daring, to suggest that it has done so in a seemingly deliberately adaptive way, in order to ensure its own continued existence. This sounds purposive, but Lovelock is careful to avoid the teleological trap; he assumes Gaia is the product of natural selection.*

*Just as natural selection has mindlessly moulded the behaviour of individual bees so that they maintain the hive at a common optimal temperature, it has moulded the behaviours of all the individual producers and consumers of carbon dioxide, oxygen, nitrogen and methane, and all of the organisms whose activities can influence global climate and oceanic salinity, so that these parameters will be maintained within ranges hospitable to life as a whole…*

*…Lovelock is not explicit, but he implies that Gaia evolved as life evolved and if he is to be taken seriously at all, he must mean that she is the product of natural selection, operating in the normal way but on a grand scale. And that must mean that organisms (or at least those with important geochemical impact) which behave in such a way as to contribute to the maintenance of Gaia have a greater probability of leaving offspring than those which do not.*

In response to Doolittle’s review, Lovelock appealed to the importance of emergent phenomena in climate, and Margulis emphasized under-appreciated sources of variability during Darwinian evolution [1]. However, neither of Gaia’s advocates directly addressed Doolittle’s key concern, the question of whether organisms that ‘contribute to the maintenance of Gaia’ somehow have a greater time-integrated relative fitness than those that do not. At face value (and as pointed out by both Doolittle [1] and later Dawkins [3]), the answer to this question is seemingly so obviously ‘no’ that the fact that the Gaia hypothesis makes it necessary to ask such a question suggests that it is predicated upon a fundamental misunderstanding of Darwinian evolution.

The classic Darwinian objection to Gaia has been repeated throughout the literature for decades and runs roughly as follows. Natural selection involves the non-random differential survival and reproduction of a subset of the variation initially present in a biological population [4]. Thus, selection requires a varying population of comparable entities in order to operate, with the combination of heredity, variation and selection typically regarded as a ‘recipe’ for the occurrence of Darwinian evolution [5]. The operation of selection results in an increase in the proportion of a population exhibiting heritable characteristics that positively impact survival and reproduction (fitness). Such characteristics are necessarily those conducive to the processes of growth, survival and reproduction, meaning that selection’s long-term action explains the unique congruence between living organisms and their environments [6]. Neither the Earth-system as a whole, nor any of the planetary/climatic scale entities to which the Gaia hypothesis refers, exist in an interacting population of comparable entities conforming to this recipe (heritable variation causing differential survival and reproduction). Therefore, whatever properties that climatic feedback and biogeochemical cycles may have, these properties cannot be the direct result of natural selection.

However, the debate over the Gaia hypothesis’s legitimacy did not end with this difficulty, in large part because Gaia’s existence is supported by a credible [7–9], albeit debatable [10], interpretation of the evidence. Lovelock’s initial inspiration was his sense that the far-from-equilibrium composition of Earth’s atmosphere, qualitatively distinct from the ‘dead’ atmospheres of comparable lifeless planets such as Mars or Venus, would prove to be the definitive observational marker for the presence of life in the universe [11,12]. If a representative sample of Earth’s atmosphere was placed in a sealed container with a representative sample of surface rock, then left for a geologically representative time interval (say, 10 000 years) the composition of both would change considerably via various reactions. If the same experiment were repeated with a Martian atmosphere and rock, the sample’s composition would remain approximately the same. This reflects how dynamically sustained atmospheric chemical disequilibrium remains credible as a definitive fingerprint for life in context of astrobiological exploration [13]. The implication of this ‘Lovelock test’ is therefore that if life is found elsewhere in the universe, it will not be (say) some bacteria in an isolated microcosm, but an entire biosphere occurring in conjunction with such atmospheric disequilibrium, and perhaps some form of coupling to planetary/climatic homeostasis. In other words, life elsewhere in the universe will not persist for long enough to be discovered, unless it gets past a ‘Gaian bottleneck’ [14].

Various additional observations are interpreted by Gaia’s advocates as best explained by some sort of life-driven homeostatic system. These include:

— continuous occurrence throughout Earth’s history of (broadly) life-permitting temperatures, allowing the ongoing presence of liquid water, despite significant variation in the intensity of solar radiation input [15],
— strong biologically driven recycling of biologically essential elements (CHNOPS and trace elements)—such that the cycling ratio (between the quantity of the element within the biosphere and its influx from outside sources) vastly exceeds that which would be expected without life [16,17], and
— the operation of various stabilizing negative feedbacks on the level of oxygen [18], greenhouse gas concentrations [19] and the level of key nutrients [20] in the ocean and atmospheric components of the Earth system.

For critics, these properties can also be interpreted as ‘nothing but’ byproducts of life’s presence, in context of the boundary conditions provided by the Earth-system. To some extent, this is merely a difference of interpretation. For instance, the macronutrient composition of marine surface waters generally remains near to Redfield N : P stoichiometry, an observation that has received detailed attention from Gaia’s strongest advocates [20] and harshest critics [21]. The relevant feedbacks result from N : P co-limitation, combined with the fact that nitrogen fixers are the only organisms capable of growth in N-depleted waters. The question is whether explaining the observations requires more than the general idea that life affects and is affected by its environment. The existence of an entire taxonomy of Gaia-variants (emphasizing luck, byproducts, life-environment coevolution and homeostasis), is interpreted by some critics as indicative of a vagueness about the hypothesis’s core predictions [22].

Habitability refers to a set of conditions sufficient for the long-term survival and flourishing of life, thus pertains to a *planetary average* state, sufficient for the long-term persistence of *life as a whole*. Gaia’s focus on planetary scale habitability unavoidably entails averaging over the extensive variation between species in environmental preferences, so as to think in terms of the boundary conditions permissive for life in the universe, conceived more broadly. It also necessitates grappling with the challenging and unresolved [23,24] question of life’s definition. Realistically, life is probably distinguishable from non-life by a list of characteristics rather than a single essential property, perhaps because the difference is one of degree rather than of kind [25]. Individual living organisms exhibit properties such as metabolism, internal stability via the external production of entropy, and physiological control. Populations of living organisms exhibit the information-based properties (heredity, variation, selection) that define Darwinian evolution. Ambiguous edge cases result when these control-based and information-based properties are decoupled. A flame produces entropy. Minerals grow and exhibit complex organization. Negative feedbacks and dynamically maintained steady states are also found in non-living systems (e.g. the Oklo reactor [26]). A computer virus can undergo Darwinian evolution of a sort, but a collection of sterile hybrids cannot; and so forth. Notwithstanding these definitional subtleties, pragmatic adoption of NASA’s operational definition of life, as ‘a self-sustaining chemical system capable of undergoing Darwinian evolution’ (https://astrobiology.nasa.gov/research/life-detection/about/) [27] covers the above duality between physiological control and information propagation. It is also lends itself to a similarly pragmatic definition of what habitability amounts to: the combined presence of a solvent (probably liquid water), a free energy source, a permissive temperature window and availability of Carbon, Hydrogen, Nitrogen, Oxygen, Phosphorus, Sulphur (CHNOPS) and trace elements [28].

Given these introductory considerations, here I will understand the Gaia hypothesis in the only sense that (to my mind) unequivocally distinguishes the idea from a more generic life-environment coevolution:

*The Gaia hypothesis proposes the existence on Earth of a system of dynamically maintained chemical disequilibrium that is planetary in scale, habitability promoting, and causally connected to the presence of life. A prediction that follows from this hypothesis is that any sustained presence of life elsewhere in the universe will necessarily occur in conjunction with an analogous system.*

This is a strong version of the Gaia hypothesis that would count within the taxonomy of Gaia-variants as something like a ‘probable homeostatic Gaia’ [29]. I argue that in order to be more than a generic appeal to the existence of various feedbacks in planetary sciences, Gaia must be life-specific and relativized to comparable lifeless planets. In order be distinguishable from a more general view of life-environment coevolution, Gaia must be habitability promoting. In order to be unambiguously falsifiable, Gaia must go hand-in-hand with the prediction that if life is found elsewhere in the universe it will be in conjunction with planetary-scale chemical disequilibrium.

This understanding of Gaia demands a clear resolution of the problem posed by Doolittle in 1981, which here I will label the ‘question of a Darwinized Gaia’ (QDG):

*QDG: is there any conceivable way in which, on average and over the entirety of Earth’s history, habitability promoting genotypes tend to have a greater time-integrated fitness than genotypes that destroy habitability (or have a neutral effect)?*

Embedded within this question is the idea of a ‘habitability promoting genotype’. This is not a straightforward concept, given the above-mentioned need to average over between species variation in environmental optima in order to scale up to planetary habitability. Nevertheless, assuming that habitability in general is a coherent concept (not all planets in the universe will support life), a habitability-promoting genotype can be informally understood as one with a geochemical/climatic effect that promotes the long-term planetary scale persistence of life as a whole. In other words, a habitability promoting genotype is one with a phenotype that results in what Wilkinson has termed a ‘Gaian effect’ [30] upon the Earth system. Crucially, this implies that a habitability promoting genotype need not have any particular impact (positive or negative) on its own immediate environment or relative fitness. This reflects the separation of spatial and temporal scales between planetary climatic habitability and Darwinian evolution, which in turn distinguishes the Gaia problem from adjacent concepts such as niche constructionism (whereby organisms/genotypes modify their environments in a way that has a knock-on effect on their relative fitness [31]).

It is thus informative to cross reference the veracity of the Gaia hypothesis against the perceived answerability of the QDG. Three categories of interpretation suggest themselves.

## 2. Options for answering the question of a Darwinized Gaia

### (a) Option 1: the Gaia hypothesis is incorrect, and the question of a Darwinized Gaia is redundant but probably answerable in the negative

Gaia’s critics, as mentioned, argue that Earth’s biogeochemical properties are not ‘geophysiological’, but best explained by interactive coevolution between life and its environment. In a detailed critical examination [10] of the evidence for and against Gaia, Tyrell argues:

— atmospheric chemical disequilibrium does not require homeostatic regulation, merely that life affects its environment,
— the correspondence [32] between the N : P stoichiometry of the oceans and the bodies of marine phytoplankton can be explained by feedbacks acting on the boundary conditions for growth of different forms of life, and is not analogous to intra-organism homeostasis,
— the geochemical and climatic impact of evolutionary innovations has been at least as likely to impede the flourishing of life as promote it,
— the observed relationship between temperature and life (basal metabolic rates, total community biomass and taxonomic diversity) generally conforms to a unidirectional causal dependency in which ‘temperature paces life’, rather than any interactivity,
— a slightly warmer Earth with higher CO_2_ levels would probably increase global net primary productivity, suggesting that neither of these variables is subject to an optimizing regulation, and
— Earth’s surface temperatures have not been regulated within a habitable range, as evidenced by events such as the Proterozoic global scale glaciations, and the decisive factor allowing for life’s persistence across Earth’s history is a strong element of luck.

In this light, it should also be noted that a credible argument can be made that life, especially complex life, is at least as likely to drive itself extinct by undermining the conditions allowing it to flourish, as it is to perpetuate habitable conditions [33]. Gaia’s advocates would, of course, strongly debate each of these points. Where critics argue that global scale glaciations falsify Gaia in the negative, advocates claim that such events indicate that the efficiency of temperature regulation has itself improved over time, and that imperfect regulation does not indicate the complete absence of regulation. Then again, if life’s ‘flourishing’ on Earth (putatively increasing global productivity and diversity, declining extinction rates) supports the case for the increasing effectiveness of homeostatic mechanisms over time [34], does the Earth’s being too cold for maximal primary production, or the occurrence of past extinction events, count against Gaia’s existence? How exactly should the overall tally be calculated?

The evidence for failure of various regulatory mechanisms during Earth’s history, and the corresponding need to invoke some degree of luck in explaining the long-term persistence of habitability [35], can be framed in terms of the anthropic principle. This refers to an inherent restriction on the range of possible observations that can be made about the universe, imposed by the fact that observations are only possible under conditions that permit the appearance of intelligent observers. In this context, the evidence for Gaia can arguably be subsumed into anthropic observer bias: had habitable conditions not been sufficiently persistent to permit complex life and intelligent observers, we would not be present to ask the question [36,37]. On this view, asking questions like: ‘why has the Earth remained sufficiently habitable to support the emergence of complex observers?’ is akin to asking: ‘why do I always see roads when I go driving?”.

While undeniable, this principle does little to mechanistically explain the difference between Earth and comparable lifeless planets, at best demanding the reformulation of a ‘why?’ question into a ‘how?’ question. In other words, an anthropic perspective shows that it is somewhat confused to ask *why* the Earth has remained sufficiently habitable to allow the appearance of complex observers, given that we are such observers. However, this does not negate the question of exactly *how* Earth is different from a hypothetical alternative version of itself that did not support the emergence and persistence of habitability.

Dismissal of Gaia as simply false has the advantage that the disconnect from Darwinian evolution highlighted by the QDG ceases to be a problem. However, it leaves unanswered the reciprocal question of the net impact of natural selection on Earth’s climatic stability. If Gaia is simply rejected, there is no clear answer to seemingly fundamental questions like ‘does life, and in particular the occurrence of Darwinian evolution by natural selection, make the Earth more or less stable?’.

### (b) Option 2: the Gaia hypothesis is plausibly correct, even though the question of a Darwinized Gaia is answerable in the negative, because natural selection is not of direct relevance to producing any homeostatic regulation

Acutely aware of the above ‘Darwinism problem’, Gaia’s contemporary advocates have tended to distance themselves from Lovelock’s initial likening of the Earth to an organism [34], and suggested various non-Darwinian habitability-promoting influences, as well as factors that may mitigate against natural selection’s potential favouring habitability destroying genotypes. The general idea is that Gaia is somehow caused by life but not by selection. One way to interpret this view is in context of the above-mentioned edge cases concerning the definition of life: Gaia is a planetary scale manifestation of a subset of life’s physiological and cybernetic properties (control, negative feedback, resistance to perturbation), decoupled from life’s information-based properties. (While future biosphere-level reproduction [38] cannot be ruled out, it cannot be invoked to explain Earth’s current properties or biogeochemical history). This option has received the most attention within the Gaia-associated literature.

#### (i) Physical constraints on adaptation

An integral controller exerts an influence proportional to the integral (as opposed to a single time-specific value) of the error (deviation from the target value) exhibited by the controlled variable. Integral rein control refers to the presence of two integral controllers simultaneously acting on the same variable in opposing but complementary directions (i.e. one increasing the controlled variable if it falls too low, the other decreasing it if it rises too high). A characteristic feature of the integral rein control common within the physiology of living organisms is zero steady-state error [39]. One example is the regulation of blood sugar by both insulin and glucagon. In theory, having one of these hormones either produced, or not, in proportion to blood sugar’s deviation from optimum, would have some regulatory effect. However, the adaptive value of zero steady-state error plausibly explains why the body uses two hormones in conjunction. Fascinatingly, the concept of integral rein control, and its application, by mathematician Peter Saunders, to the regulation of blood sugar using two opposing hormones [39,40] was inspired by the Gaia hypothesis.

The famous Daisyworld model [41] was developed by Watson and Lovelock to defend Gaia against accusations of teleology and inconsistency with Darwinian evolution. Daisyworld is inhabited by one black and one white daisy species and is subject to a linearly increasing temperature forcing. The two species share an optimum growth temperature but alter their environment in opposite but complementary ways; blacks warm their immediate environment, whites cool theirs. This is owing to a difference in the fraction of incoming radiation reflected by organisms within each species, i.e. an environmental byproduct of organism physiology. If the planetary average temperature is too warm, the white species (the local temperature of which is cooler than planetary average) exhibits increased growth, which cools Daisyworld. Similarly, if Daisyworld is too cold the black species gains a growth advantage, warming the planet. This leads to temperature homeostasis despite increasing insolation forcing, illustrating how unconscious biological growth could give rise to integral rein control with zero-error regulation.

Although Daisyworld is typically interpreted as an illustrative parable, some argue that there is evidential support for the operation of integral rein control in Earth’s climate system. Leggett and Ball propose a topological similarity between temperature, CO_2_ and pressure time series data and an integral control system [42], and go on to argue that similar datasets support the notion that the biosphere is the locus of this controller [43]. However, the more fundamental problem with Daisyworld corresponds precisely to the strength of the analogy with blood sugar regulation. The functional specificity of the latter is an evolutionary adaptation derived from natural selection, whereas Daisyworld’s integral rein control must be built into the underlying assumptions of the model. The magnitude of the growth advantage gained by one species over the other is proportional to that of the temperature’s deviation from optimum (integral control). Which species gains a growth advantage fortuitously corresponds to the one that will bring the system back towards optimum (two opposing and complementary reins). Thus, and as was quickly pointed out by critics [44], local natural selection might just as plausibly produce a destabilizing influence (e.g. a white daisy species with a colder optimum temperature for growth than the black species will cool the planet, derive a growth advantage from this cooling, therefore cool the planet further, inducing a positive feedback that undermines habitability).

One way out of this problem is to note the growth functions of real organisms are bounded, such that growth falls off away from the optimum state in a manner described by a hat function. The existence of fixed constraints on the boundary conditions within which biological growth can occur may mean that no species can ‘push’ a climatic variable in a particular direction indefinitely, and such destabilizing influences will self-limit before inducing complete loss of habitability [45]. This concept can be expanded into a hierarchical view, within which biological growth and changes in the abundance of different phenotypes, can be subsumed into the totality of positive and negative Earth-system feedbacks, from which habitable conditions emerge [46]. It is undeniable that physical constraints impose restrictions on where life can exist and how genetic innovation can change phenotypes while retaining viable organisms. However, this cannot be a comprehensive solution to the puzzle of Gaia and the QDG. If the persistence of habitable conditions is entirely the consequence of physical constraints on growth and adaptation, it is (by definition) impossible for life to drive itself extinct. Under such circumstances Gaia is rendered superfluous.

#### (ii) Habitability is a byproduct not an adaptation

Lynn Margulis famously remarked that Gaia could not be an organism because no organism consumes its own waste [47], reflecting how organisms exist in an environmental context in which they are surrounded by the physiological byproducts of other organisms. The concept of a ‘byproduct’ is life-specific, an output of a process that is causally incidental to the main function of that process for the organism. The byproducts of growth need not be selected for or against, nor must they come with an energetic cost. Volk explores the idea that Gaia is an emergent property resulting from the totality of the byproducts of life [16,17], persuasively arguing that the powerful nutrient recycling observed in Earth’s oceans can be explained in this way.

Perhaps the byproducts of life simply increase the variety of chemical reactions that are present within the Earth system, and homeostasis/habitability emerges as a result. Ashby’s principle of ‘requisite variety’ states that when a system is responding to an external disturbance, the variety in the outcome cannot be less than the ratio between the variety in the disturbance and that in the system itself [48]. More simply, this means that during active regulation, a system must have sufficient variety within its range of potential control responses to match the variety in the external stimulus that initially destabilizes it. From this perspective, perhaps life’s diversification increases the variety of responses to perturbation that the biosphere and/or Gaia can exhibit. However, this implicitly makes the long-debated and contentious [49] assumption that the presence of diversity is more likely to produce stability than destroy it. There is no clear justification for the idea that the average effect of life’s physiological byproducts should have any systematic impact on habitability, any more than the products of abiotic processes.

#### (iii) Gaia is an example of a general stability producing tendency exhibited by complex systems

A statistical description of thermodynamic entropy refers to the disorder or ‘mixed-up-ness’ of a system composed of multiple constituent parts. A macroscopic state (macrostate) refers to a plainly observable top-down property of a system and is comprised of multiple microstates (precise details of the position and momentum of individual components). The entropy of a macrostate is proportional to the natural logarithm of the number of possible microstates corresponding to that macrostate. A high entropy macrostate is one corresponding to (i.e. potentially being composed of) a larger number of microstates than a comparable low entropy macrostate. For example, when a cloud of gas molecules spreads out across a fixed space from a single source, the aggregate shape of the cloud is analogous to a macrostate, with a single molecule in a single position corresponding to a microstate. A macrostate involving the cloud’s being confined to a single corner of the space corresponds to fewer microstates than does the same cloud’s being spread out more evenly. A direct analogue of thermodynamic entropy is used in information theory, in which the information entropy quantifies the uncertainty associated with the state of a random variable [50].

The principle of maximum entropy production states that processes operating far from thermodynamic equilibrium tend to reach steady states at which the dissipation of energy and the production of entropy occurs at a maximum rate [51]. Planetary scale entropy production is highly likely to be a strong marker for any extra-terrestrial biospheres [52]. From this perspective, several models of ‘entropic Gaia’ argue that long-term interaction between life and its abiotic environment results in a state of increasing information entropy (characterized by increased biomass and taxonomic diversity, as well as self-perpetuating life-environment interactions [53]). The question, therefore, becomes that of how and why any such entropic state relates to habitability.

A related concept is sequential selection, whereby life’s impact on habitability goes through multiple resets [54], eventually leading to improved regulation. Given a model system in which there is heritable variation within the biosphere in terms of environmental impact, it is imaginable that habitability may be promoted by sequential selection, assuming some kind of ratcheting mechanism can be postulated whereby the susceptibility to complete collapse is reduced with each reset event [53]. Despite featuring the word ‘selection’, sequential selection is not a Darwinian mechanism, although it may occur in systems containing Darwinian populations. Sequential selection is transformational (acting on the properties of a single entity) rather than variational (acting on the composition of a population of entities with varying properties) [55]. However, the assumption that such a ratcheting mechanism exists, as noted in this issue [55], appears again to amount to the dubious ‘diversity begets stability’ proposition.

Importantly, if sequential selection’s ratcheting mechanism is interpreted as pertaining to information entropy in general, the mechanism need not require biology at all [34]. This might explain the persistence of habitable conditions, but by the same measure would erode Gaia’s life-specificity and invite the question of why analogous homeostatic processes do not arise on comparable lifeless planets. More fundamentally, why does each of the reset events that sequential selection invokes not irreversibly undermine habitability? If this is assumed to be impossible then sequential selection presupposes the above view that physical constraints on adaptation prevent life driving itself extinct—and Gaia is again redundant. If not, then sequential selection requires a degree of luck that increases multiplicatively with each reset invoked.

### (c) Option 3—The Gaia hypothesis is plausibly correct and the question of a Darwinized Gaia may be answerable in the affirmative, in context of a 'Darwinized’ Gaia

In recent years, Ford Doolittle has reconsidered his initial critical assessment of the Gaia hypothesis and attempted to begin deriving a Darwinized version [56,57], involving an understanding of natural selection that does not require replicating entities. However, some would argue that any analogue of selection without replication is a pale imitation of the conventional process [58]. Absent the iterative occurrence of replication with mutation generating novel heritable variation, selection is potentially restricted to stability-based sorting [59] within bounds defined by the variation initially present in the population of interest, and such that the total amount of variation present can only decrease. On the other hand, persistence based selection has recently received increased attention from philosophers of biology, keen to assess the extent to which these concerns can be mitigated [60,61].

An informative introductory example to persistence selection is provided by clade selection. A clade is a monophyletic taxonomic group containing an ancestor and all its descendants, meaning that, by definition, a clade cannot replicate. Some clades persist longer than others, and clade longevity is a function of the number and specific evolutionary ecology of the constituent species. Propensity towards speciation is plausibly attributable to species level properties (geographical range, ecological and phenotypic diversification, population patchiness, etc.). Thus, persistence selection can differentiate between clades by virtue of their propensity towards internal taxonomic diversification [25], which in turn results from the properties of their member species [62]. The concern with this line of reasoning, as with the iconic case of species selection [63], is that any persistence selection of large scale entities is epiphenomenal, i.e. comprehensively explained as a byproduct of lower-level replication-based selection of genotypes/organisms in the taxa contained within the clade. On some level, this same concern arises in context of the QDG.

Doolittle’s ‘its-the-song-not-the-singers’ (ITSNTS) theory proposes differentially persisting, non-replicating variants of biogeochemical cycles and the genotypes that sustain them [64,65]. ‘Recruitment’, by biogeochemical cycles, of the sets of genotypes that interconvert distinct species of the relevant element/nutrient (nitrate and ammonium, sulphide, sulphate, etc.), leads to a closing of the cycle. Continuous re-association between such genotypes and the nutrients/growth substrates on which they depend constitutes ‘re-production’ of the cycle-biota interaction, a process-level regeneration of the collective ‘song’, without replication in the conventional evolutionary sense. The taxa (singers) lead to the collective existence of the cycle (song), and the cycle’s existence perpetuates the niches that these taxa require. Crucially, ITSNTS proposes that this situation is more than just epiphenomenal because persistence selection makes a difference to the genetic composition of the biosphere: novel alleles/genotypes arise and increase from rarity via natural selection, by virtue of an interaction with a geochemical cycle, such that absent past persistence selection for this interaction, these biological novelties would not arise, because the relevant taxa would be absent or fundamentally different.

In principle, ITSNTS marks the first step towards a view in which at least asking the QDG is no longer unthinkable. It is uncontentious that global biogeochemical cycles exhibit a historicity, and that evolutionary innovations may have climatic effects, which may be dramatic. However, the testability of this view is a more subtle question. There arises the question of exactly what kind of entity ITSNTS-style persistence selection acts upon, and how such entities can be identified in nature. The answer to questions of this sort can be interpreted in two different ways.

#### (i) Biogeochemical cycles are interactors, and biogeochemical cycles are (maximally) extended phenotypes

A replicator is an entity that passes on its structure across generations, most obviously a genetic sequence that leaves a copy of itself in the next generation. An interactor is an entity that interacts with its environment as a cohesive whole (most obviously an individual organism with a developmental history, an ecology and physiology) and which manifests the phenotype corresponding to the relevant replicator [66]. With reference to this ‘replicator-interactor framework’ the totality of microbes that sustain a particular global biogeochemical cycle might be designated a ‘persister’, which interacts with its ‘fitness landscape’ (climate and geochemistry) via the cycle in question. Synergistic interactions between genes in (say) nitrogen fixers and denitrifiers arise by mutual reinforcement of the relevant niches, via the ongoing recycling of this element in the world’s oceans. This might be interpretable as a Gaian (or at least planetary scale) extension of the concept of an ‘extended phenotype’ associated with Dawkins’s gene centric view of evolution [67]. Virtually all the biogeochemical cycling on which ITSNTS focuses is microbially mediated, with many steps involving redundancy whereby different taxa perform the same ‘geophysiological’ function, often via lateral gene transfer, which lends itself to the gene centric view. However, this view is not so much an answer to the QDG as a framing of planetary scale properties in terms of more traditional gene-centrism.

#### (ii) Persistence selection between ‘cycle-biota variants’

Multi-level selection (MLS) theory deals with forms of biological organization subject to replicator dynamics at different spatial and temporal scales, with incompatible equilibrium solutions [68,69] (e.g. cancer within multicellular organisms dependent upon controlled internal cell growth). Within the lexicon of MLS, individual ‘cheaters’ outcompete ‘altruists’, but groups of altruists outcompete groups of cheaters. Which category of phenotype prevails in real systems depends upon population structure, kin relatedness, evolutionary shared interest between individuals and the relative rates of the dynamics at different levels.

The process-level persistence selection invoked by ITSNTS can emerge in models as a form of group viability selection (groups of altruists survive better than groups of cheaters) for elemental recycling [70]. In an environmental context in which the availability of the relevant element is scarce, but this element is nevertheless essential for survival, only organisms associated with the cycle will survive. Furthermore, an environmental context of this sort may also partially address persistence selection’s variation-supply problem, via net genetic assimilation of ancestrally facultative physiological traits. That is, a recycling phenotype is generated by genetic mutation in the normal way, but interaction with the cycle selects for expression changes, such that the phenotype transitions from an environmentally sensitive trait to a constitutive or ‘hard wired’ one.

Perhaps the most fundamental problem relating to ITSNTS is deriving an objective definition of the sort of entities that differentially persist relative to one another. Any biogeochemical cycle consists of multiple interconversions between distinct variants of an essential nutrient, in a cross-linked network-like structure. Where does one cycle (song) begin and another end? One way to answer this question may be with reference to a single biologically facilitated pathway for net recycling of an essential nutrient, plus the totality of genotypes driving the relevant interconversion reactions, *if any only if this pathway is associated with a unique climatic effect that influences its relative persistence* [71]. Conventional biological individuals are defined by a reliable coupling between genotype and phenotype (replicator and interactor, heritable variation, fitness, etc.). Similarly, the kind of entities that differentially persist within ITSNTS can be meaningfully defined as a coupling between ‘internal’ properties (genotypes and reactions) and external geochemical and climatic impact ‘phenotypes’. Specifically:

*A cycle-biota-variant (CBV) is definable as the coupling between (a) one pathway for the net recycling of a biologically essential nutrient, plus the genotypes driving the relevant interconversion reactions, and (b) a long-term, planetary scale climatic/geochemical effect uniquely attributable to this pathway that affects its relative persistence.*

Say there are two distinct CBVs (that is, two topologically distinct pathways for net recycling of the same nutrient) $CBV_{1}$ and $CBV_{2}$. Suppose $CBV_{1}$ induces a unique environmental effect α. Suppose further that effect α interacts with independent Earth-system feedbacks f(α,X) to elicit directional (i.e. irreversible) change X. If effect X negatively affects $CBV_{2}$ relative to CBV, then competition between different CBVs on the basis of unique climatic ‘phenotypes’ has the potential to induce irreversible changes in allele frequencies analogous to allele fixation or ecological competitive exclusion [71].


$$CBV_{1}\overset{\text{causes}}{\to }\alpha \overset{\text{triggers}}{\to }f\left(\alpha ,X\right)\overset{\text{results in}}{\to }X\overset{\text{causes}}{\to }\left(\frac{CBV_{1}}{CBV_{1}+CBV_{2}}\approx 1\right).$$

 Far from being ad hoc, this general trajectory is observed multiple times during the numerous biogeochemical revolutions that characterize Earth’s geochemical history. Most obviously, during the aftermath of the great oxidation, various geochemical cycles featured the displacement of cycle-variants associated with low oxygen boundary conditions, by equivalent variants stable under a high oxygen state [71]. The irreversibility of climatic effect "X" plausibly exemplifies the numerous instances of bistability and hysteresis (history dependence) observed within the climate system, which can lead to irreversible transitions in Earth’s biogeochemical state after the crossing of a threshold, referred to within the literature on contemporary climate change as "tipping points" [72].

Importantly, this sort of evolutionary trajectory makes CBV-level persistence selection qualitatively different from conventional ecological interactions. In specific regard to the QDG, the directional climatic feedback f(α,X) may occur at any spatial or temporal scale, thus be decoupled from Darwinian evolution. The key difference between this kind of CBV level persistence selection and (say) niche construction or interference competition is the extra degree of freedom provided by the scaling up to climate (the knock-on feedback effect f(α,X) resulting from the immediate biologically induced effect α). This introduces a ‘historicity’ (potential for irreversibility) and will involve climatic processes that are random with respect to the internal properties of the CBV itself. (The latter arguably invokes a loose parallel with the randomness of mutation with respect to its evolutionary effects [71]). In simulations exhibiting the above sort of trajectory covariance patterns between these internal (genotypes and reactions) and external (climatic effects) CBV properties can observationally track persistence selection—in just the same way that covariance between fitness and traits under normal Darwinian selection tracks conventional Darwinian dynamics.

CBV-level persistence selection therefore provides a potential framework to answer the QDG in roughly the way proposed by Doolittle in his ITSNTS theory. However, the nature of any such future answer remains unclear. One CBV’s displacing a competitor by inducing an environmental effect does not relate, at least with any clarity, to global-scale habitability for life as a whole. However, it does entail the perpetuation of conditions that are habitable for the surviving dominant metabolisms.

## 3. Conclusions

I began this essay by defending the idea that if the Gaia hypothesis is to be substantive and falsifiable it must invoke the existence of a system that is planetary in scale, a consequence of life’s presence, habitability promoting and present elsewhere in the universe (via planetary atmospheric chemical disequilibrium as a reliable marker for life). I then argued that this definition of Gaia unavoidably requires a clear answer to Doolittle’s original concern about the relative fitness of habitability promoting genotypes, which here I labelled the question of a Darwinized Gaia (QDG). I then divided attempts to evaluate and develop Gaia into ‘reject’, ‘accept without Darwinizing’ and ‘Darwinize’. Table 1 shows the relationship between different interpretations of Gaia relative to these categories, along with relevant unanswered questions.

**Table 1.** The status of different categories of non-selection focused explanations for the observations motivating the Gaia hypothesis.

interpretation		planetary scale entity?	habitability-promotion?	life-specificity?	outstanding issues/questions
the Gaia hypothesis is probably incorrect and a misinterpretation of the evidence		no	no	no	is life-in-the-universe a planetary phenomenon? Is atmospheric chemical disequilibrium relevant to extra-terrestrial life detection?
Earth’s habitability reflects anthropic observer self-selection bias		no	no	yes	how does Earth differ from a hypothetical version of itself that does not permit the emergence of complex life and observers?
physical constraints on adaptation limit the spread of habitability destabilizing phenotypes		no	yes	yes	if taken to be the entire explanation for Earth’s habitability, renders Gaia superfluous
byproduct Gaia		yes	unclear	yes	why should the byproducts of life be more conducive to maintenance of habitability than the products of abiotic chemical reactions on lifeless planets?
sequential selection	via the biosphere	yes	yes	yes	why doesn’t each reset irreversibly destroy habitability? Ratcheting mechanism reduces to dubious claim that ‘complexity begets stability’
	via a general information-entropy related constraint	yes	yes	no	does this mechanism operate on lifeless planets?
Darwinized Gaia (ITSNTS or equivalent)	maximally extended phenotype	yes	unclear	yes	questionable testability, concern of ‘nothing-but-ism’
	persistence selection	yes	yes, for some cycle-biota-variants	yes	promotes differential impact of some cycle biota variants, not universal habitability

The basic arguments that I have made here can be summarized as follows.

(i) there is no need to Darwinize Gaia if she does not exist. However, those who reject Gaia ignore the planetary/climatic evidence supporting her existence, as well as fundamental questions like ‘What is the effect of natural selection on Earth’s stability?’;
(ii) the anthropic principle is relevant to habitability, but simply restates the necessity of Earth’s observer permissiveness, as opposed to explaining the difference from comparable lifeless planets;
(iii) physical constraints on growth and adaptation are similarly relevant to the persistence of habitability, but if such constraints are the entirety of the explanation, then it is impossible for life to drive itself extinct, which makes Gaia redundant;
(iv) if the byproducts of life’s presence promote habitability in a way that the products of abiotic processes would not, then Gaia could coexist with a negative answer to the QDG. However, it is unclear why this should be the case;
(v) sequential selection necessarily assumes that no single reset event permanently drives all life extinct, and that habitability promotion improves with each reset, rather than demonstrating why this is the case;
(vi) any framing of Gaia in terms of maximum entropy production reduces her life specificity, begging the question of why the relevant processes do not operate on comparable lifeless planets;
(vii) a fully Darwinized Gaia is an imaginable future extension of Doolittle’s ITSNTS theory, raising the previously unthinkable possibility that Gaia may exist in conjunction with the QDG’s being answerable in the affirmative; and
(viii) persistence selection of distinct variants of biogeochemical cycles may occur in a unique way if such variants exhibit climatic-impact ‘phenotypes’ that feed-back on their relative persistence.

Overall, the current balance of evidence and theoretical modelling allows each of the above options 1−3 to be reasonably defended and prevents the QDG from being answered unequivocally. However, thanks to Doolittle’s recent pioneering work, this question can be framed in far more concrete terms than was previously possible. Theoretical expositions may help clarify the ambiguous relationship between Gaia’s veracity and amenability to Darwinization. However, the constraints imposed by the sample-size-of-one problem probably means that these questions will only be decisively answered by exoplanet exploration.

## Data Availability

This article has no additional data.
